# Hemoperitoneum Secondary to Spontaneous Rupture of a Retroperitoneal Varix

**DOI:** 10.1155/2017/1829676

**Published:** 2017-08-03

**Authors:** Derrick D. Eichele

**Affiliations:** Divisions of Gastroenterology & Hepatology, University of Nebraska Medical Center, 982000 Nebraska Medical Center, Omaha, NE 68198-2000, USA

## Abstract

Hemoperitoneum due to a ruptured retroperitoneal varix is an exceedingly rare condition and a poor prognostic sign with catastrophic and life-threatening complication of portal hypertension. We present a unique case of a 56-year-old female with cirrhosis secondary to primary sclerosing cholangitis who presented with acute abdominal pain and hypovolemic shock prior to a cardiac arrest following a ruptured retroperitoneal varix without prior esophageal varices and a newly identified intrahepatic cholangiocarcinoma. The clinical presentation with abdominal pain and hemorrhagic shock is consistently reported in the relevant literature. Early recognition affords appropriate management and urgent surgical intervention leading to survival.

## 1. Introduction

Variceal hemorrhage is a common complication of portal hypertension with reduction in mortality in recent decades secondary to early identification and surgical management. Spontaneous hemorrhage from ruptured retroperitoneal varices is a rare event with less than thirty-five reported cases in the literature since 1958 [1–6]. The clinical presentation with abdominal pain, rapid distension with syncope, and hypotension is nearly universal. Survival potential is contingent upon early diagnosis with paracentesis leading to emergent laparotomy with varix plication.

## 2. Case Report

**Figure 1:**
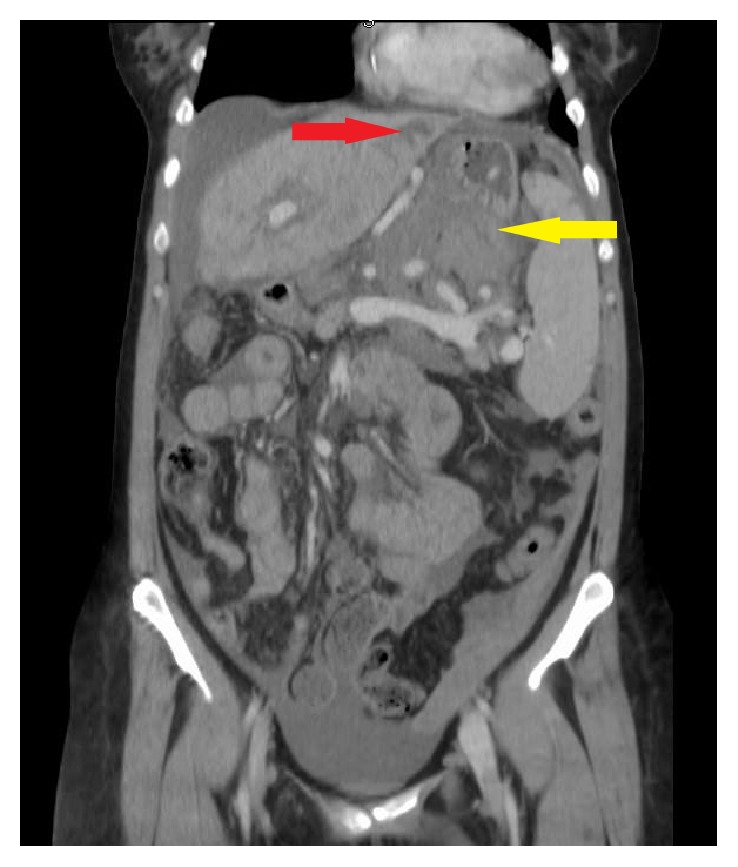
Coronal image from abdominal CT with contrast captures the development of hemoperitoneum (yellow arrow) from splenic varices and hypodensity in left lobe of liver suggestive of cholangiocarcinoma (red arrow).

A 56-year-old female with cirrhosis secondary to primary sclerosing cholangitis without prior varices presented with acute onset of intense abdominal pain and dizziness. Pertinent presenting vitals and laboratory values included a systolic blood pressure of 60 mmHg; hemoglobin, 6.8 g/dL; platelets, 101,000/mm^3^; INR, 1.4; creatinine, 1.2 mg/dL; albumin, 1.6 g/dL; and bilirubin, 1.2 mg/dL. Initial resuscitation included three liters of normal saline (NS), two units of packed red blood cells (PRBC), and two units of fresh frozen plasma (FFP). Hemodynamics were stable following resuscitation and therefore computed tomography (CT) was performed (Figure 1). The report indicated hemoperitoneum with a large hematoma (12.8 × 6.8 × 16 cm), multiple splenic hypodensities suggestive of infarct, umbilical, and perisplenic varices, and a left hepatic lobe hypodensity not present in magnetic resonance cholangiopancreatography (MRCP) performed six months earlier. Following admission to the intensive care unit, the patient again became hypotensive and sustained pulseless electrical activity (PEA). Adult cardiac life support (ACLS) was initiated and patient was resuscitated and endotracheally intubated. Emergent exploratory laparotomy revealed active bleeding from a retroperitoneal varix on the inferior border of the pancreas; it was ligated and oversewn resulting in cessation of bleeding. The liver lesion was identified and needle biopsy was performed prior to abdominal closure. Intraoperatively the patient received an additional 8 units of PRBC, 8 units of FFP, 3 units of platelets, 500 ml of albumin 5%, 1200 ml from cell saver, and 1 liter of NS. Blood loss was estimated at 4 liters. Postoperative laboratory values included hemoglobin, 11.3 g/dL; INR, 1.3; creatinine, 1.23 mg/dL; AST, 326 IU/L; ALT, 134 IU/L; and bilirubin, 1.5 mg/dL. The patient remained hemodynamically stable following the immediate postoperative period. In the succeeding days the patient developed worsening multiorgan failure with shock liver (AST, 2439 IU/L; ALT, 1322 IU/L, and bilirubin, 5.4 mg/dL) and acute renal failure (creatinine, 4.63 mg/dL) resulting in initiation of continuous venovenous hemodialysis (CVVHD). The liver biopsy was reported as a well-differentiated adenocarcinoma of the liver, most consistent with cholangiocarcinoma. Due to the grim prognosis, the family elected for comfort care measures. The patient expired on the 5th day of hospital admission due to multiple organ failure.

## 3. Discussion

Portal hypertension in cirrhosis develops from the resistance to blood flow and diversion away from the liver through collateral circulation to low-pressure systemic vessels. Resultant increase in intravenous pressures may lead to the development and dilation of portosystemic collateral circulation. The anastomoses between portal and systemic circulations occur at locations in which veins draining into the dual systems are juxtaposed and are commonly encountered at the gastroesophageal junction, anorectal plexus, and umbilical vein [4, 7, 8]. In addition, varices also develop where organs supplied by the splanchnic circulation come in contact with the retroperitoneum [9].

Historically, trauma and nonmalignant gynecological conditions account for more than 90% of intra-abdominal hemorrhage, whereas hemorrhagic ascites is encountered in only 5% of cirrhotic patients [1, 10]. Spontaneous hemoperitoneum in cirrhosis usually develops from an identifiable lesion such as hepatocellular carcinoma, ovarian carcinoma, rupture of intraabdominal varices, and hemorrhagic pancreatitis [10, 11].

In nearly all instances, rupture of intra-abdominal varices has been encountered in the setting of cirrhosis with evidence of coexisting portal hypertension and esophageal varices. Prior variceal hemorrhage or variceal band ligation has not been present [2, 4, 5, 10, 11]. The true incidence of retroperitoneal varices is difficult to estimate, but in one series the incidence was 18% on abdominal CT, but only a limited number of cases have been reported in the literature [12]. Clinical presentation is found with near uniform consistency with abdominal pain presentation documented in two-thirds of all cases as well as syncope and hypotension [6]. The intensity in which abdominal pain is present is related to the rapidity and volume of blood extravasation into the peritoneum [10]. Although rapid abdominal distension is often encountered, peritoneal signs are usually absent.

The diagnosis has been established via paracentesis, angiography, ultrasound, computed tomography, or laparotomy. Literature notes that CT identifies the retroperitoneal varices more reliably than angiography [12, 13]. Of the six reported patients that underwent angiography to diagnose and ligate the bleeding vessel, in only one case was the vessel identified and ligated, but this did not lead to survival [6]. Ultrasonography has been deemed an insensitive modality to detect small retroperitoneal varices [13, 14]. The diagnosis of hemoperitoneum made by paracentesis is evident by ascitic fluid hematocrit greater than 5% and may exceed peripheral blood concentration [2, 10]. In all survival cases, early paracentesis was performed leading to emergent laparotomy. The only documented therapy with survival benefit is operative exploration with plication of the bleeding vessel, whereas nonoperative treatment did not lead to survival in any case [2, 4, 6, 11]. Mortality in all cases has ranged up to 78%, but in a recent estimate that employed emergent operative management to control the bleeding vessel mortality improved to 57%. The mortality risk was felt to be resultant to overwhelming hypovolemic shock at presentation, but this report lacked inclusion of initial resuscitation measures during diagnostic evaluation in its suggested flowchart to optimize treatment [6, 11].

Spontaneous hemoperitoneum in cirrhotic patients is a poor prognostic sign as it is associated with increased risk of hepatorenal syndrome and high mortality rate [1, 10]. Unique to this case was the lack of prior identified esophageal varices on routine surveillance endoscopy or imaging from six months prior to this presentation. Additionally, the patient had the newly identified intrahepatic cholangiocarcinoma not identified with MRCP. Initial resuscitation appeared appropriate, but delayed laparotomy likely reduced the chance of survival. The significance of hemorrhagic shock on presentation and early operative intervention with control of the bleeding source are known to effect survival, whereas portosystemic shunting remains speculative [2, 6, 10, 11, 15].
